# Photovoltaic road pavements as a strategy for low-carbon urban infrastructures

**DOI:** 10.1016/j.heliyon.2023.e19977

**Published:** 2023-09-09

**Authors:** Giulia Del Serrone, Paolo Peluso, Laura Moretti

**Affiliations:** Department of Civil, Constructional and Environmental Engineering, Sapienza University of Rome, Via Eudossiana 18, 00184, Rome, Italy

**Keywords:** Urban heat islands, Photovoltaic pavement, Greenery, Cool pavements, Road energy harvesting, ENVI Met, DIALux, Life cycle cost

## Abstract

Urban Heat Islands (UHIs) are expanding in anthropized areas, causing serious climatic consequences such as rising temperatures and citizens' discomfort. Several studies have identified and confirmed how the use of cool road pavements can mitigate and reduce the negative effects of UHIs. This study performs a microclimate simulation of San Pietro in Vincula Square in Rome through ENVI-Met software by replacing the current asphalt pavement in the parking area with a cool one. The proposed layout consists of light concrete pavers in the parking lots, parking aisles made of photovoltaic (PV) panels, and a perimeter hedge. The innovative use of PVs is analysed from the thermal and economic viewpoints alike. In the first case, its thermal characteristics like those of asphalt provide results in terms of air temperature, mean radiant temperature, and predicted mean vote close to the current ones. Furthermore, the energy analysis shows the PV's effectiveness in terms of economic impact. Indeed, the electricity produced by the proposed PV system is enough to light the area, and its surplus can power public activities such as electric vehicle charging. The initial investment would pay for itself in the 25-year service life as confirmed by the positive net present value (NPV), and the cash flow reveals a break-even point in the 15th year.

## 1 Introduction

In recent years, climate change has been spreading globally and showing all its negative and irreversible consequences [1]. The anthropization of the natural environment and urbanisation of rural areas contributed to the growing global warming [2]. Moreover, incorrect urban planning favoured the formation and diffusion of the phenomenon of Urban Heat Islands (UHI) [3]. Indeed, the major issues of modern cities (e.g., buildings' size, surfaces' colours, population density, and low vegetation cover) affect the urban microclimate [4] and energy consumption [5]. UHI's air temperatures (ATs) are 1 °C–10 °C higher than those of rural areas [6,7] due to impermeable building materials. Indeed, materials of roads, parking lots, and roofs [8] have high thermal capacity so, when monitored for 24 h [9], it has been observed that they absorb heat during the day and release it during the night [10], with a maximum temperature difference of 20 °C and 4 °C during the day and night periods, respectively [11]. In the scientific literature, several strategies are proposed to counteract and mitigate the UHI effects [12,13] through reflective, porous, stone, and grass surfaces instead of asphalt ones. Cool pavements [14,15] account for up to 5 °C reduction in AT [16,17], up to 20 °C reduction in mean radiant temperature (MRT) [18], and increased well-being in terms of Predicted Mean Vote (PMV) [19]. Another strategy implies Urban Green Infrastructures (UGI), *vertical* greenery systems, or renovation of walls and roofs. Several studies demonstrated that green roofs combined with photovoltaic (i.e., PV) panels can reduce AT by almost 3 °C [20,21]. Nevertheless, photovoltaic canopies guarantee a reduction of 13.2 °C in AT over the shaded pavement compared to the adjacent fully exposed one [22]. Several studies proposed solar or photovoltaic panels to mitigate UHIs [23] in line with the global strategy towards a low-carbon economy. This approach encourages the strategy of generating renewable and sustainable energy from streets and surrounding public spaces [24,25] to be used for various mobility-related applications, such as infrastructure lighting [26], speed data collection [27], communication between vehicles [28], and EV charging stations [29].

In these cases, the road space consumption becomes a resource for the installation of photovoltaic panels [30] to be embedded into the infrastructure (e.g., noise barriers [31], solar arches [32] and canopies [22]). In other cases, however, the photovoltaic panels become an integral part of the road structure, generating electricity and supporting traffic loads [33,34]. Thermal investigations have found photovoltaic pavements allow for a 5 °C decrease in the surface temperature and a 1 °C decrease in the AT [35]. The life cycle cost analysis indicated that the high initial costs are recovered through the revenues of renewable energy production and the additional social and environmental benefits, such as the reduction of 2.15 × 10^7^ kg of CO_2_ emissions over 1 km for a four-lane road during its life cycle [36].

According to the Italian standard for the promotion of the minimum environmental criteria (CAM) [37], this study investigates a sustainable layout to improve the microclimate conditions of an existing square in Italy and answer the current efforts in energy transition [38]. In previous studies, the authors investigated San Pietro in Vincula Square, in the historic centre of Rome, and demonstrated the effectiveness of cool pavements in terms of AT reduction and the well-being perceived by citizens [39,40]. In particular, the most recent research [41] proved that concrete pavers, surrounding greenery, and trees imply significant mitigation of UHI with an AT reduction higher than 3 °C, half MRT value, and a maximum PMV value equal to 2.2. Due to the historical and cultural heritage of the investigated site, the authors proposed the construction of photovoltaic pavements instead of photovoltaic canopies to reduce the visual impact of the energy and microclimate strategies. According to the interesting results reported in Ref. [41], concrete block pavers are designed in the stall areas, photovoltaic panels are laid in the parking lot aisles, and UGIs are along the boundary. The software ENVI-Met [42] is used to model and analyse the microclimate performance of the square in July 2022. The results in terms of AT, MRT, and PMV allow a critical and unbiased comparison of the proposed layout with the existing one. Finally, the energy and economic balance of the installation and maintenance works of the panels, lighting costs, and electricity revenues during a 25-year service life permit an assessment of the effectiveness of the investigated solution. With respect to the previous works, this study confirms that photovoltaic road infrastructures can produce sustainable electricity for public lighting, but they can also be a new source of income, selling the surplus energy for recharging electric vehicles.

## 2 Materials and methods

**Fig. 1:**
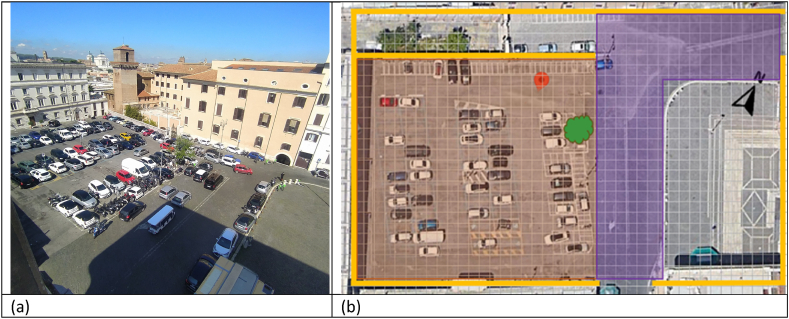
San Pietro in Vincula Square: (a) top view; (b) site map.

This study presents the microclimate analysis of San Pietro in Vincula Square (Fig. 1a) in Rome (lat. 41°53′36.8″–long. 12°29′32.1″). Currently, the area has two types of pavement: asphalt on the parking surface (Orange area in Fig. 1a) and modular stone pavement on the carriageway (Purple area in Fig. 1 b-).

Innovative pavement materials have been introduced and investigated to evaluate the best ATs, MRTs, and PMVs results. In the literature, several studies demonstrated the pivotal role of the thermal properties of cool building materials (such as albedo and thermal inertia [43], emissivity, thermal conductivity, and heat capacity [44]) in the mitigation of UHIs [45]. Albedo and emissivity are the main parameters affecting the thermal performance of road pavements. The former is the ability to reflect incident radiation, the latter is the ratio of the energy radiated from a material's surface to that radiated from a blackbody under the same boundary conditions; both range between 0 and 1. Table 1 lists the performance of the investigated pavement materials.

**Table 1:** Physical characteristics of the pavement materials.

Material	Albedo (−)	Emissivity (−)
Asphalt	0.2	0.9
Basalt pavers (Sampietrini)	0.4	0.9
Light Concrete	0.8	0.9
Photovoltaic Panels	0.1	0.9

**Fig. 2:**
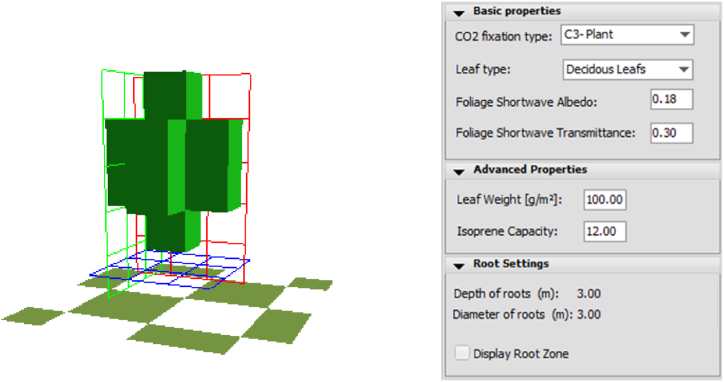
Tilia tomentosa properties in a 3D ENVI Met model.

The current layout is poor in vegetation because there is only one Tilia tomentosa 5 m-high tree with a small trunk (Fig. 2) between the parking area and the carriageway, while the proposed solution has a 1-m-high boundary Buxus sempervirens hedge dense. Table 2 lists the greenery's height values and thermal properties [46].

**Table 2:** Physical characteristics of the green elements.

Element	Height (m)	Albedo (−)	Transmittance (W/m^2^K)
(Existing) deciduous spherical tree	5	0.18	0.3
(Added) Buxus sempervirens hedge dense	1	0.20	0.3

**Fig. 3:**
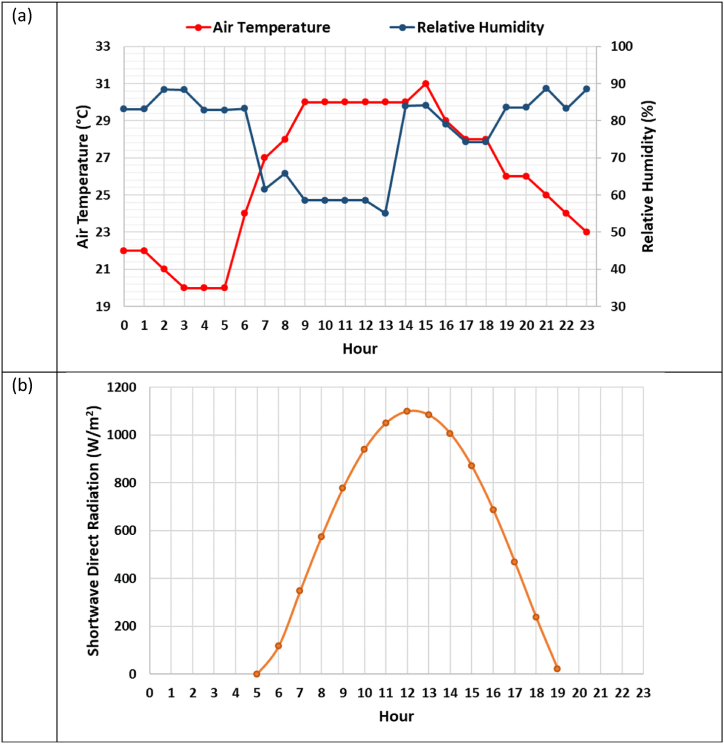
Weather conditions: (a) Daily profile of AT and RH; (b) Daily profile of shortwave direct radiation.

The microclimate analyses have been performed using the ENVI-Met software because it can simulate urban thermal conditions, as demonstrated by other studies in the literature [42,47]. It consists of a 4-level structure. The first level involves the definition of input data (i.e., a three-dimensional -3D-model of the survey area, weather data, road surface, soil type, and vegetation). The second level performs the microclimate analysis, while the third one delivers the output files. Finally, in the last level, Leonardo and ASCII tools allow reading and analysing the results using two-dimensional (2D) coloured maps or binary output files, respectively [48,49]. The geometric dimensions and resolution of the spatial domain, the weather input data, and the survey period have been modelled according to Table 3. The survey was carried out on July 18, 2022, one of the hottest days in the last summer. The air temperature (AT) and relative humidity (RH) in Fig. 3 a come from official weather archives [50]. Each simulation lasts 72 h until the software reaches numerical stability and dissipates the spin-up effect.

**Table 3:** Initial and boundary conditions used in ENVI-Met simulations.

Parameter	Definition	Value
Computational domain and grid	Grid cells	40 × 30 × 30
	δx × δy × δz	2 × 2 × 2 m
Weather conditions	Wind speed	3.0 m/s
	Wind direction	West
	AT	Daily profile July 18, 2022 (Fig. 3a)
	RH	Daily profile July 18, 2022 (Fig. 3a)
	Shortwave direct radiation	Daily profile July 18, 2022 (Fig. 3b)
Simulation Time	Start Date	July 16, 2022
	Start of simulation	00:00 h
	Total simulation time	72 h

**Fig. 4:**
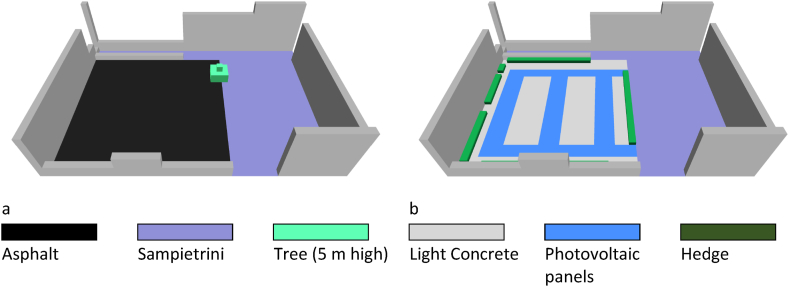
Scenarios simulated with ENVI-Met: (a) First Scenario (AS); (b) Second Scenario (LC + PV).

Fig. 4 shows the simulated scenarios:

1. the first scenario (AS) recreates the current layout, where a flexible pavement covers the parking area (Fig. 4a);
2. the second scenario (LC + PV) consists of a photovoltaic pavement for the parking aisles, a concrete block pavement for the parking lots, and a boundary hedge.

In this study, the thermal analysis involved AT, MRT, and PMV, allowing quantitative and qualitative evaluations of physical and comfort thermal conditions [51,52]. In particular, MRT assesses the effects of ambient radiation on the human body [53,54] according to Equation (1):

(1) $${MRT}^{4}=\sum_{i=1}^{N}T_{i}^{4}F_{p\to i}$$

where i ranging from 1 to the N surrounding surfaces, $T_{i}$ is the temperature of the surrounding surfaces, and $F_{p\to i}$ are view factors between the test subject and the whole surrounding environment.

PMV is a human comfort index [55] based on physical and physiological parameters that correlate with thermal comfort and weather parameters [56]. In this study, the reference man is 35 years old and 1.75 m-tall, and he weighs 75 kg. The PMV results are interpreted according to the ASHRAE scale [57]. In detail, negative and positive values are related to cool and warm sensations, respectively, where ideal, acceptable, and critical conditions are identified as follows:

• −1≤PMV≤1 Ideal Conditions (IC);
• −3<PMV<−1∨1<PMV<3 Acceptable Conditions (AC);
• PMV≤−3∨PMV≥3 Critical Conditions (CC).

In addition to the microclimatic approach, this study also investigates the energy impact of solar pavements. Road pavements absorb abundant solar radiation, and photovoltaic panels can turn it into energy, becoming an energy harvesting solution [58,59]. It is composed of three main layers, among which the solar cell is the core electric element. There is the surface transparent layer, the middle functional layer, and the bottom protective one [30].

The interactive tool PhotoVoltaic Geographical Information System (i.e., PVGIS) of the EU-Joint Research Center in Ispra has been used [60] to estimate the average monthly and yearly energy production (E_p_/m^2^) of a PV system with crystalline silicon cells connected to the electricity grid. The adopted solar radiation database with hourly time resolution is PVGIS-SARAH2 (0.05° × 0.05°), produced by the Satellite Application Facility on Climate Monitoring (i.e., CM SAF). The value of the assumed peak power of the PV array under standard test conditions is equal to 0.142 kWp (kilowatt-peak), according to the market average values declared by manufacturers. The tool can also estimate the losses due to temperature and irradiance effects, as well as losses in cables, power inverters, and dirt on the modules, through the system loss percentage. Moreover, the modules that are composed of crystalline silicon can suffer a physiological power decrease during their life cycles. For this reason, a constant total of 22% of system loss has been introduced in the tool. Due to the application of the PV modules on the road, the building-integrated option with little or no air movement behind the modules has been chosen, with an inclination angle from the horizontal plane equal to zero. Then, the energy production of the entire photovoltaic system has been calculated by multiplying the m^2^ value by the total solar pavement area.

**Fig. 5:**
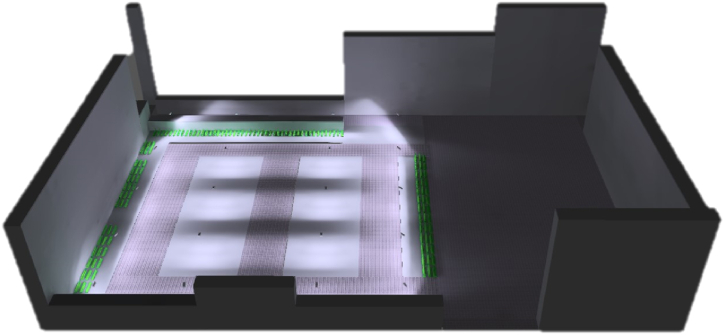
3D model of San Pietro in Vincoli Square in DIALux.

A redesign of the existing public lighting in the studied area has been proposed to benefit from the sustainable energy stored through the solar pavement. The lighting design of the parking area was carried out with DIALux software, freely available from the DIALux Website [61]. The software initially involves a detailed three-dimensional reconstruction of the project area, with special attention given to the pavement type because its materials’ reflectance affects the lighting performance [62]. Fig. 5 shows the 3D model of the LC + PV scenario in DIALux; also, the current AS scenario has been modelled.

The lighting design of the parking area was carried out following the standard UNI EN 12464-2 [63]. The project includes the installation of 20 streetlights, equipped with Gewiss GWR5773B LED lamps, whose lighting data are in Table 4.

**Table 4:** Gewiss GWR5773B lighting data.

	Photometric curve	Luminaire luminous flux (ff)	lm
		11000	
		Power connection (P)	W
		77	
		Efficiency (Ef)	lm/W
		142.9	

The energy absorption (E_A_) is the lamp power connection (P) multiplied by the switch on time (t), which varies during the solar year according to Ref. [64].

The overall life cycle cost due to the installation and maintenance of the manufacturing panels is according to Equation (2):

(2) $${\text{CP}}_{\text{TOT}}={\text{CP}}_{i}+\sum_{t=1}^{N}Cm\left[\frac{{\left(1+i\right)}^{t}}{{\left(1+r\right)}^{t}}\right]$$

where CP_TOT_ is the discounted panels cost, CP_i_ is the panel installation cost (1627.5 €/m^2^), and C_m_ is the manufacturing maintenance cost assumed to be equal to 1000 € in the construction year.

**Fig. 6:**
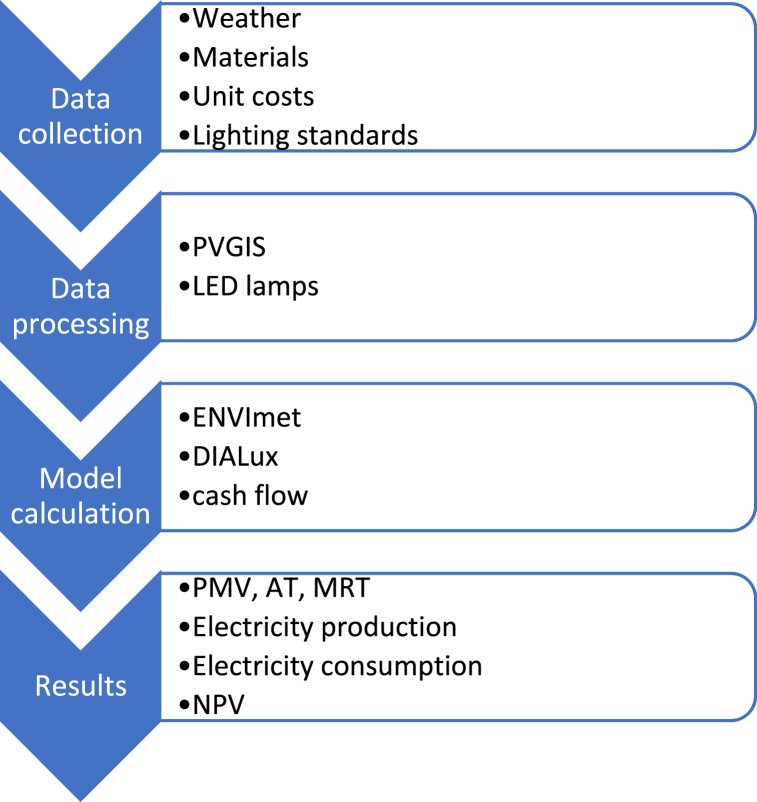
Flow diagram.

In this study, the comparison between the manufacturing discounted costs and the revenues from electricity production allowed an optimisation of the public investment to pursue social, environmental, and economic benefits. Fig. 6 shows the flow diagram of this study.

## 3 Results

**Fig. 7:**
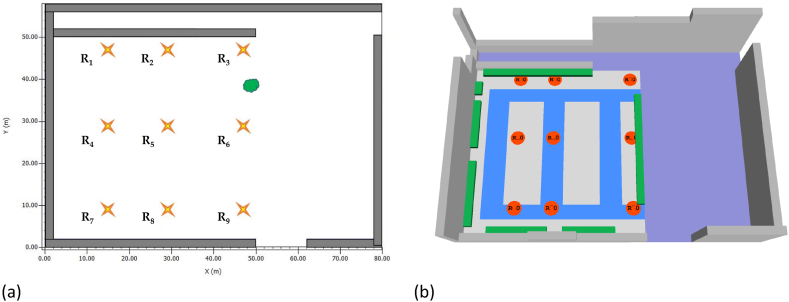
Simulated model in ENVI-Met and location of receptors: (a) 2D visualisation; (b) 3D visualisation.

The results section is divided into two macrotopics, the first of which addresses the analysis of the thermal response of the square to different pavement types and the second of which addresses the evaluation of the energy benefits from photovoltaic panels. For a quick and localised investigation of the square, 9 receptors (R_i_, i = 1, …,9) were included in the simulated model. Fig. 7 shows the simulated model in 2D (Fig. 7a) and 3D (Fig. 7b) visualisation.

### 3.1 Thermal analysis

#### 3.1.1 At results

For both AS and LC + PV scenarios, a statistical 24-h analysis of AT was performed. Based on the data collected from the 9 receptors, maximum, minimum, and average AT values (T_max_, T_min_, and T_avg_, respectively) and their standard deviations (σ) were evaluated at each hour. Table 5 and Table 6 show the AS and LC + PV results for the hottest hours of the design day.

**Table 5:** Air temperature from 9:00 a.m. to 6:00 p.m. on July 18, 2022 - scenario AS

	AT (°C)									T_max_	T_min_	T_avg_	σ
Hour	R_1_	R_2_	R_3_	R_4_	R_5_	R_6_	R_7_	R_8_	R_9_	(°C)	(°C)	(°C)	(°C)
9 a.m.	29.39	29.50	30.07	28.98	29.01	29.28	28.69	28.67	28.82	30.07	28.67	29.16	0.45
10 a.m.	30.34	30.50	31.23	30.00	30.03	30.42	29.77	29.73	30.08	31.23	29.73	30.23	0.46
11 a.m.	30.95	31.14	31.99	30.64	30.68	31.17	30.47	30.42	30.85	31.99	30.42	30.92	0.48
noon	31.36	31.58	32.52	31.09	31.14	31.69	30.97	30.92	31.38	32.52	30.92	31.40	0.49
1 p.m.	31.50	31.77	32.74	31.27	31.38	31.97	31.20	31.20	31.69	32.74	31.20	31.63	0.49
2 p.m.	31.43	31.74	32.69	31.22	31.39	31.99	31.20	31.25	31.76	32.69	31.20	31.63	0.48
3 p.m.	31.75	32.05	32.94	31.33	31.63	32.19	31.26	31.45	31.92	32.94	31.26	31.84	0.52
4 p.m.	30.83	31.09	31.84	30.30	30.96	31.52	30.41	30.94	31.46	31.84	30.30	31.04	0.51
5 p.m.	29.62	29.71	30.49	29.32	29.74	30.46	29.42	29.84	30.58	30.58	29.32	29.91	0.48
6 p.m.	28.83	28.97	29.44	28.75	29.06	29.49	28.76	28.93	29.59	29.59	28.75	29.09	0.33

**Table 6:** Air temperature from 9:00 a.m. to 6:00 p.m. on July 18, 2022 - scenario LC + PV.

	AT (°C)									T_max_	T_min_	T_avg_	σ
Hour	R_1_	R_2_	R_3_	R_4_	R_5_	R_6_	R_7_	R_8_	R_9_	(°C)	(°C)	(°C)	(°C)
9 a.m.	28.77	28.84	29.20	28.36	29.01	28.72	28.55	28.67	28.82	29.20	28.36	28.77	0.24
10 a.m.	29.57	29.69	30.17	29.21	29.99	29.67	29.54	29.67	29.88	30.17	29.21	29.71	0.28
11 a.m.	30.08	30.24	30.76	29.70	30.57	30.28	30.14	30.27	30.55	30.76	29.70	30.29	0.31
noon	30.50	30.74	31.18	30.05	30.97	30.78	30.56	30.68	31.05	31.18	30.05	30.72	0.34
1 p.m.	30.62	30.88	31.35	30.20	31.16	30.97	30.76	30.89	31.26	31.35	30.20	30.90	0.35
2 p.m.	30.58	30.84	31.30	30.09	31.12	30.96	30.63	30.85	31.24	31.30	30.09	30.85	0.38
3 p.m.	30.91	31.16	31.68	30.32	31.29	31.16	30.63	30.97	31.32	31.68	30.32	31.05	0.40
4 p.m.	29.80	30.00	30.50	29.47	30.53	30.34	29.63	30.35	30.69	30.69	29.47	30.15	0.43
5 p.m.	28.77	28.90	29.35	28.61	29.07	29.35	28.63	28.98	29.74	29.74	28.61	29.04	0.37
6 p.m.	28.26	28.36	28.62	28.13	28.54	28.59	28.07	28.17	28.74	28.74	28.07	28.38	0.24

Whatever the pavement type, in Table 5, Table 6 the receptors detect different hourly values that are not far apart. Indeed, σ varies between 0.24 °C and 0.52 °C. Therefore, from now on the analysis of the results will focus on R_4_, R_5_, and R_6_, because they fall on the light concrete pavers, on the photovoltaic pavement, and near the perimeter hedge, respectively (Fig. 7b). Table 7 compares the ATs of the two scenarios at noon (i.e., the time when the sun is at its zenith) and at 3:00 p.m. (i.e., the hottest hour).

**Table 7:** AT values at noon. and 3:00 p.m.

Scenario	AT (°C) at noon.			AT (°C) at 3:00 p.m.		
	R_4_	R_5_	R_6_	R_4_	R_5_	R_6_
**AS**	31.09	31.14	31.69	31.33	31.63	32.19
**LC** + **PV**	30.05	30.97	30.78	30.32	31.29	31.16
**ΔAT (°C)**	1.04	0.17	0.91	1.01	0.34	1.03

**Fig. 8:**
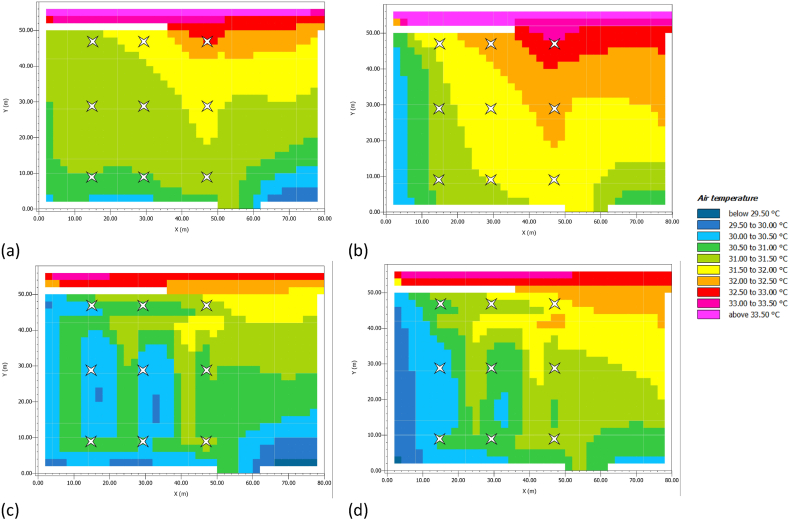
AT in July. (a) AS at noon.; (b) AS at 3:00 p.m.; (c) LC + PV at noon; (b) LC + PV at 3:00 p.m.

Table 7 highlights how the proposed LC + PV strategy advances a lowering of AT. In detail, compared to AS there is a reduction of about 1 °C on concrete pavement and near the hedge, while the difference is minimised on PV pavements (i.e., 0.17 °C). Fig. 8 a-d shows the 2D coloured map of the AT trend at noon and 3:00 p-m. for AS and LC + PV, respectively.

Fig. 8 confirms the benefits of reducing AT through LC + PV compared to AS. Both at noon and 3:00 p.m., Fig. 8 c and 8.d shows a lower AT in the parking area. In addition, the colour trend highlights the higher contribution of concrete pavers compared to PV panels.

#### 3.1.2 MRT results

The survey continues with an evaluation of MRT for the same time and receptors considered for AT (Table 8).

**Table 8:** MRT values at noon and 3:00 p.m.

Scenario	MRT (°C) at noon			MRT (°C) at 3:00 p.m.		
	R_4_	R_5_	R_6_	R_4_	R_5_	R_6_
**AS**	62.75	62.56	62.58	45.51	62.39	62.40
**LC** + **PV**	66.85	66.61	66.48	48.19	64.67	64.38
**ΔMRT (°C)**	−4.10	−4.05	−3.90	−2.68	−2.28	−1.98

**Fig. 9:**
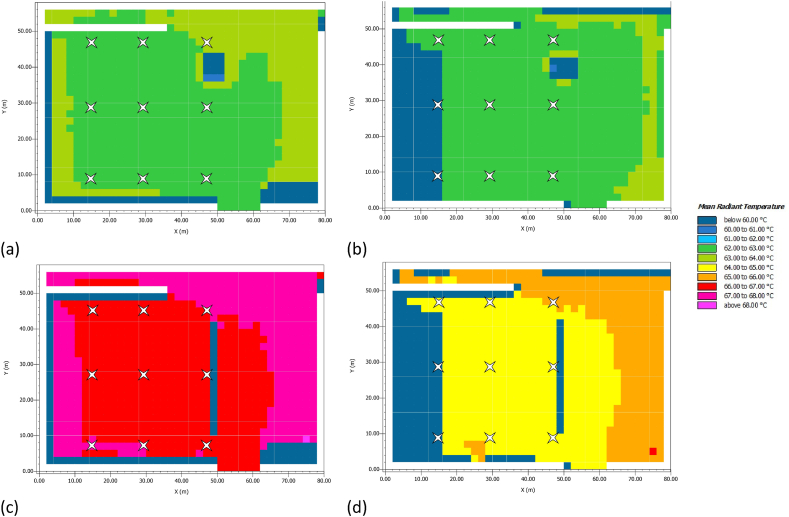
MRT in July. (a) AS at noon; (b) AS at 3:00 p.m.; (c) LC + PV at noon; (b) LC + PV at 3:00 p.m.

Table 9 finds that the worst strategy is LC + PV because it produces an increase in MRT of about 4 °C over AS. Fig. 9 shows both for AS and for LC + PV the trend of MRT throughout the survey area at noon and at 3:00 p.m., confirming the results in Table 8. The maps manifest a uniform trend of MRT, except for the perimeter areas where the shadows of the buildings and the green elements favour a reduction of the thermal parameter. Moreover, Fig. 9 highlights the beneficial contribution of greenery to human comfort because it can reduce the exchange of heat between man and the surrounding environment.

**Table 9:** PMV values at noon and 3:00 p.m.

Scenario	PMV at noon			PMV at 3:00 p.m.		
	R_4_	R_5_	R_6_	R_4_	R_5_	R_6_
**AS**	3.93	3.93	4.06	3.15	4.18	4.31
**LC** + **PV**	3.87	4.07	4.03	3.17	4.21	4.17
**ΔPMV**	0.06	−0.14	0.03	−0.02	−0.02	0.14

#### 3.1.3 PMV results

The thermal survey ends with the evaluation of the PMV index (Table 9).

**Fig. 10:**
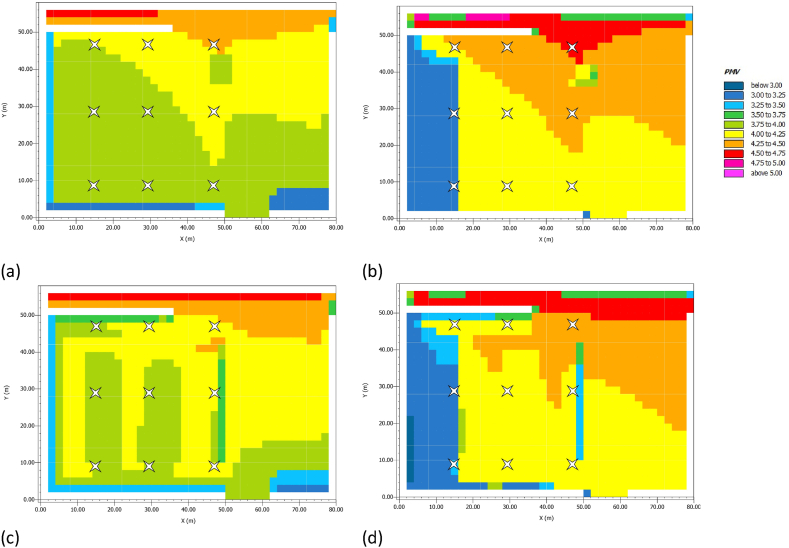
PMV in July. (a) AS at noon; (b) AS at 3:00 p.m.; (c) LC + PV at noon; (b) LC + PV at 3:00 p.m.

The last row of Table 9 indicates that the difference between the values of AS and LC + PV is close to 0, showing no great benefit from the innovative strategy. Fig. 10 shows for both AS and LC + PV the trend of PMV throughout the survey area at noon and at 3:00 p.m., confirming the results in Table 9. At a fixed hour, the trend of the PMV does not undergo great variations between the two scenarios. Furthermore, PMV is greater than 3 throughout the square, corresponding to critical heat conditions.

**Fig. 11:**
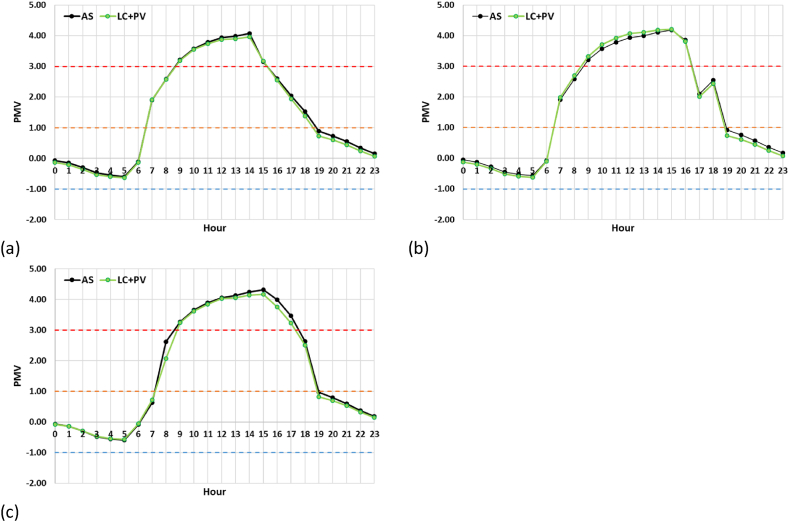
PMV in July. (a) AS at noon; (b) AS at 3:00 p.m.; (c) LC + PV at noon; (b) LC + PV at 3:00 p.m.

The high resemblance of PMV values is also recorded during the 24 h. Fig. 11a-c shows the daily profiles of PMV trends of R_4_, R_5_, and R_6_ receptors, respectively; horizontal hatching lines highlight the boundary conditions of the PMV thermal scale. For all the survey points, there is an overlap between the AS and LC + PV curves, where the time slot from 9:00 a.m. to 3:00 p.m. is marked by PMV values greater than 3 and corresponding to CC. In this study, the replacement of the current asphalt-wearing layer with light concrete pavers and photovoltaic panels did not reduce the square's hourly exposure to CC.

### 3.2 Lighting and energy benefits

**Fig. 12:**
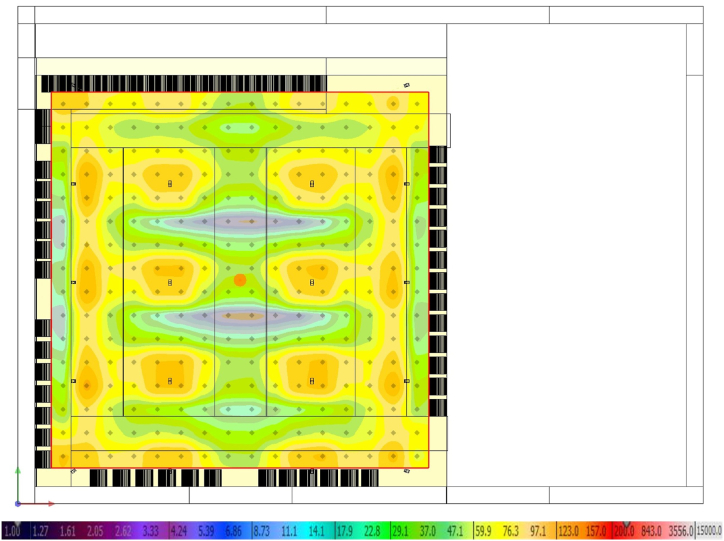
Horizontal illuminance.

The lighting redesign of the survey area has ensured an average horizontal illuminance greater than the threshold value of 10 lx required by Ref. [63]. Fig. 12 shows a 2D map of the illuminance trend in the surveyed area, whose average value is 68.1 lx. Instead, the absolute minimum is 4.9 lx, which is greater than the prescribed 2 lx value.

Table 10 shows the energy analysis output of the lighting system for each lamp:

**Table 10:** Average daily (E_A_d_) and monthly (E_A_m_) energy absorption by each lamp.

Month	Average daily switch-on time (h)	Monthly switch-on time(h)	E_A_d_ (kWh)	E_A_m_ (kWh)
January	14.3	444.3	1.10	34.21
February	13.6	379.9	1.04	29.25
March	11.8	366.3	0.91	28.21
April	9.9	297.7	0.76	22.92
May	8.5	262.0	0.65	20.17
June	7.9	236.0	0.61	18.17
July	8.3	255.9	0.64	19.71
August	9.8	302.6	0.75	23.30
September	11.5	345.8	0.89	26.63
October	13.0	401.8	1.00	30.94
November	14.6	436.5	1.12	33.61
December	15.2	471.7	1.17	36.32

The annual energy absorption (E_A_a_) by each lamp is 323.44 kWh. Therefore, the total annual amount of electricity consumed by the redesigned lightning system is 6468.76 kWh.

**Fig. 13:**
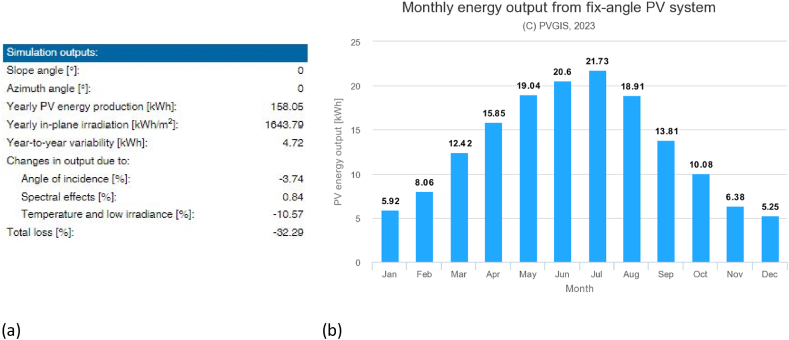
Horizontal illuminance.

On the other hand, Fig. 13 a shows the simulation outputs of the PVGIS tool for a 1 m^2^ PV panel with a total loss equal to 32.29%. Fig. 13 b, instead, presents the monthly trend of energy products by the PV per m^2^ system.

In conclusion, with a yearly PV energy production of 158.05 kWh/m^2^, the 892 m^2^ PV pavement in the redesigned scenario can produce 140,980.60 kWh of electricity annually.

## 4 Discussion

The microclimatic analysis revealed that the proposed scenario LC + PV gives benefits only in terms of AT, with a reduction of 1 °C. Besides, it is not effective to reduce MRT and ensure more comfortable conditions for PMV compared to the actual scenario AS. The geometric configuration of the proposed scenario and the thermal properties of the PV surface justify the results due to albedo and emissivity values similar to those of AS (see Table 1).

Having regard to the energy balance, the energy produced by the PV system is about 20 times greater than that required to meet the lighting needs. Surplus energy can be sent back to the supplier, with an economic return, or can be later used for public activities, like charging electric vehicles. Therefore, two comparative economic analyses have been carried out, considering the installation and maintenance of PV panels, and the electricity cash flow over 25 years. Table 11 summarises the input data for the economic assessment.

**Table 11:** Input data for the economic assessment.

Parameters	Input data	
Inflation rate [%]	i	2.7
Discount rate [%]	d	0.8
Total annual electricity consumption [kWh]	E_A_a_	6469
PV pavement unit cost [€/m^2^]	PV_UC_	1628
Total PV pavement area [m^2^]	PV_TA_	892
PV manufacturing cost [€]	PV_MC_	1451730
Total annual electricity production [kWh]	E_P_a_	140981
Solar PV export tariff [€/kWh]	ET	0.1
Electric vehicle recharging price [€/kWh]	RP	0.6

**Fig. 14:**
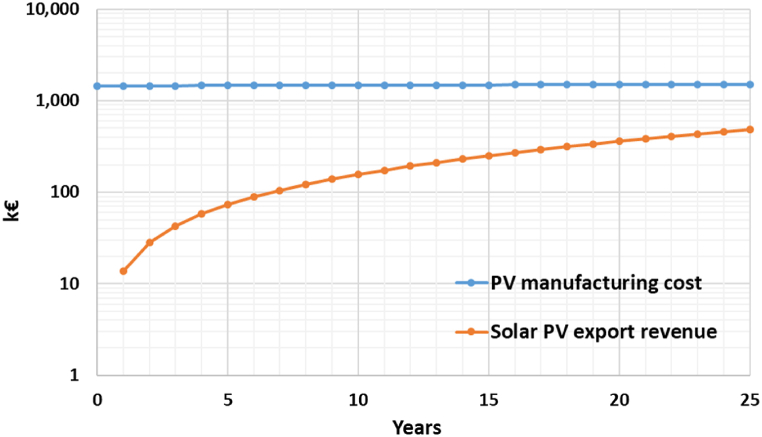
Cumulative cash flow curves over project life cycle PV manufacturing costs VS solar PV export revenues.

Fig. 14 shows the cumulative cash flow curves if all the surplus energy is sold to the grid with an ET equal to 0.1 €/kWh related to the first year.

**Fig. 15:**
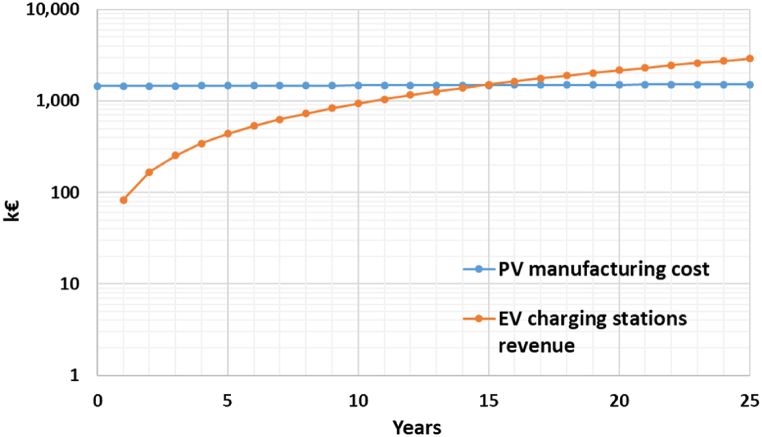
Cumulative cash flow curves over project life cycle PV manufacturing costs VS EV charging stations revenues.

Instead, Fig. 15 shows the cumulative cash flow curves if all the surplus energy is sold to EVs through the charging stations, with an RP equal to 0.6 €/kWh for the first year.

The comparison between Fig. 14, Fig. 15 highlights that the revenues from the solar PV export are not sufficient to obtain a break-even point, which is at year 15 for the option EV charging stations [30]. Besides, the calculation of the Net Present Value (NPV) confirms that only the second option is cost-effective and has a positive NPV (Table 12).

**Table 12:** NPVs of solar PV export and EV charging stations.

Options	Discounted revenues (€)	Discounted costs (€)	NPV (€)
Solar PV export	1506057	432573	−1073483
EV charging stations	1511273	2595440	1084167

While previous research has focused on just surface characteristics [65], construction [66] and maintenance costs [33], economic and energy analyses [67] of solar pavements, or on energy evaluation with solar photovoltaic carport canopy [68] and charging stations in parking lots [69], this study assesses the net present value of PV investment by focusing on two revenue alternatives between net metering and electric vehicle charging. The second option turns out to be the cost-effective solution such that a gain is guaranteed after the 15th year of service life. This result confirms the pivotal role of the impact of electricity prices on the investment decision according to Ref. [70].

## 5 Conclusion

Over the past few centuries, the economic and social opportunities offered by the cities have led people to live mainly in urban areas. Human activities and unsustainable modes of transport have increased air pollution and air temperature rise, contributing to the formation of Urban Heat Islands (UHIs).

In this study, mitigation strategies have been applied to counteract UHI in San Pietro in Vincula Square, which is a historical and architectural heritage in Rome, Italy. To this purpose, a redesign of the existing layout has been modelled through ENVI Met, replacing the actual asphalt pavement of the parking area with light concrete for the parking lots and PV panels in the aisles. Moreover, a 1 m-high boundary hedge has been added to comply with the greenery requirements laid out by the Italian Standards (CAM). Finally, a LED lighting system has been designed with the DIALux software. The innovative photovoltaic pavement is analysed from both thermal and economic viewpoints. The thermal analysis on air temperature (Fig. 8), mean radiant temperature (Fig. 9), and predicted mean vote (Fig. 10, Fig. 11) demonstrated that the proposed solution has performances comparable to those of the existing asphalt surface. In particular, the PV scenario has less than a 1 °C reduction in daily air temperature, but its diurnal MRT values reach 4 °C above the reference ones; the PMV data show no appreciable differences between the two pavements. On the other hand, more interesting are the economic results related to the sustainable energy production of the PV panels, which can be later used for public activities, including lighting the investigated area, and charging electric vehicles. The energy sale to the national grid is not profitable due to the low export tariff (i.e., 0.1 €/kWh) which implies a negative NPV (i.e., −1073483 €) in the 25 years. The loss-making option suggests providing charging stations at regular intervals in the square. Indeed, the positive NPV (i.e., 1084167 €) of this option demonstrates that the initial investment would pay for itself in the 25-year service life, and the cash flow reveals a break-even point in the 15th year.

Therefore, photovoltaic pavements in historical areas allow the urban road manager to pursue the low-carbon strategy of mobility using proper technical interventions. The results demonstrate the novelty of the proposed research by highlighting how photovoltaic road pavements can be a source of sustainable electricity for public lighting and also produce revenue through energy sales. Future studies can be carried out by applying the proposed methodology to case studies outside historic centres, where it is also possible to install photovoltaic canopies covering parking lots. In this way, the advantages coming from both net metering and a decrease in UHIs due to canopy shadows should be taken into account.

## Author contribution statement

Giulia Del Serrone, Paolo Peluso & Laura Moretti: Conceived and designed the experiments; Performed the experiments; Analysed and interpreted the data; Contributed reagents, materials, analysis tools or data; Wrote the paper.

## Data availability statement

Data will be made available on request.

## Declaration of competing interest

The authors declare that they have no known competing financial interests or personal relationships that could have appeared to influence the work reported in this paper.

## References

[1] Wit S. de, Climate S.H.-W.I.R. (2021).

[2] Fathi S., Sajadzadeh H., Sheshkal F.M., Aram F., Pinter G., Felde I., Mosavi A. (2020). The role of urban morphology design on enhancing physical activity and public health. Int. J. Environ. Res. Publ. Health.

[3] Mohajerani A., Bakaric J., Jeffrey-Bailey T. (2017). The urban heat island effect, its causes, and mitigation, with reference to the thermal properties of asphalt concrete. J. Environ. Manag..

[4] Rapti T., Kantzioura A. (2021). Study of urban microclimate conditions in a commercial area of an urban centre and the environmental regeneration potential. IOP Conf. Ser. Earth Environ. Sci..

[5] Faroughi M., Karimimoshaver M., Aram F., Solgi E., Mosavi A., Nabipour N., Chau K.W. (2020). Computational modeling of land surface temperature using remote sensing data to investigate the spatial arrangement of buildings and energy consumption relationship. Eng. Appl. Comput. Fluid Mech..

[6] Lawrence E.O., Pomerantz M., Ppn B., Akbari H., Chang S.-C. (2000).

[7] Vujovic S., Haddad B., Karaky H., Sebaibi N., Boutouil M. (2021). Urban heat island: causes, consequences, and mitigation measures with emphasis on reflective and permeable pavements. CivilEng 2021.

[8] Moretti L., Di Mascio P., Fusco C. (2019). Porous concrete for pedestrian pavements. Water 2019.

[9] Mathew A., Khandelwal S., Kaul N. (2018). Analysis of diurnal surface temperature variations for the assessment of surface urban heat island effect over Indian cities. Energy Build..

[10] Žiliūtė L., Motiejūnas A., Kleizienė R., Gribulis G., Kravcovas I. (2016). Temperature and moisture variation in pavement structures of the test road. Transport. Res. Procedia.

[11] Santamouris M. (2013). Using cool pavements as a mitigation strategy to fight urban heat island - a review of the actual developments. Renew. Sustain. Energy Rev..

[12] Akbari H., Kolokotsa D. (2016). Three decades of urban heat Islands and mitigation technologies research. Energy Build..

[13] Moretti L., Loprencipe G. (2018). Climate change and transport infrastructures: state of the art. Sustain. 2018.

[14] Nwakaire C.M., Onn C.C., Yap S.P., Yuen C.W., Onodagu P.D. (2020). Urban heat island studies with emphasis on urban pavements: a review. Sustain. Cities Soc..

[15] Ranieri V., Coropulis S., Berloco N., Fedele V., Intini P., Laricchia C., Colonna P. (2022). The effect of different road pavement typologies on urban heat island: a case study. Sustain. Resilient Infrastruct..

[16] Ko J., Schlaerth H., Bruce A., Sanders K., Ban-Weiss G. (2022). Measuring the impacts of a real-world neighborhood-scale cool pavement deployment on albedo and temperatures in los angeles. Environ. Res. Lett..

[17] Alves F.M., Gonçalves A., Enjuto M.R.D.C. (2022). The use of envi-met for the assessment of nature-based solutions' potential benefits in industrial parks—a case study of argales industrial park (valladolid, Spain). Infrastructures.

[18] Peluso P., Persichetti G., Moretti L. (2022). Effectiveness of road cool pavements, greenery, and canopies to reduce the urban heat island effects. Sustain. Times.

[19] Romano R., Bologna R., Hasanaj G., Arnetoli M.V. (2020). Adaptive design to mitigate the effects of UHI: the case study of piazza togliatti in the municipality of scandicci. Smart Innov. Syst. Technol..

[20] Teng J.W.C., Soh C.B., Devihosur S.C., Tay R.H.S., Jusuf S.K. (2022). Effects of agrivoltaic systems on the surrounding rooftop microclimate. Sustain. Times.

[21] Zhu Z., Zhou D., Wang Y., Ma D., Meng X. (2021). Assessment of urban surface and canopy cooling strategies in high-rise residential communities. J. Clean. Prod..

[22] Golden J.S., Carlson J., Kaloush K.E., Phelan P. (2007). A comparative study of the thermal and radiative impacts of photovoltaic canopies on pavement surface temperatures. Sol. Energy.

[23] Efthymiou C., Santamouris M., Kolokotsa D., Koras A. (2016). Development and testing of photovoltaic pavement for heat island mitigation. Sol. Energy.

[24] Gholikhani M., Roshani H., Dessouky S., Papagiannakis A.T. (2020). A critical review of roadway energy harvesting technologies. Appl. Energy.

[25] Pultarova T. (2017). News briefing: energy - welcome to the world's first solar road. Eng. Technol..

[26] Hu M., Song X., Bao Z., Liu Z., Wei M., Huang Y., Hu M., Song X., Bao Z., Liu Z. (2022). Evaluation of the economic potential of photovoltaic power generation in road spaces. Energies.

[27] Cantisani G., Del Serrone G., Peluso P. (2022). Reliability of historical car data for operating speed analysis along road networks. Sci.

[28] Del Serrone G., Cantisani G., Peluso P. (2023). Speed data collection methods: a review. Transport. Res. Proc..

[29] Caines A., Ghosh A., Bhattacharjee A., Feldman A. (2021). The grid independence of an electric vehicle charging station with solar and storage. Electron.

[30] Li S., Ma T., Wang D. (2023). Photovoltaic pavement and solar road: a review and perspectives. Sustain. Energy Technol. Assessments.

[31] Nordmann T., Froelich A., Goetzberger A., Kleiss G., Hille G., Reise C., Castello S. (2020). *Sixteenth European Photovoltaic Solar* Energy Conference.

[32] Trials Of Renewable Energy In Highways – The Tech Journal Available online: https://thetechjournal.com/green-tech/trials-of-renewable-energy-in-highways.xhtml (accessed on 31 January 2023).

[33] Hu H., Vizzari D., Zha X., Roberts R. (2021). Solar pavements: a critical review. Renew. Sustain. Energy Rev..

[34] Kehagia F., Mirabella S., Psomopoulos C.S. (2019). Solar pavement: a new source of energy. Bitum. Mix. Pavements VII- Proc. 7th Int. Conf. Bitum. Mix. Pavements, ICONFBMP.

[35] Efthymiou C., Santamouris M., Kolokotsa D., Koras A. (2016). Development and testing of photovoltaic pavement for heat island mitigation. Sol. Energy.

[36] Zha X., Qiu M., Hu H., Hu J., Lv R., Pan Q. (2022). Simulation of structure and power generation for self-compacting concrete hollow slab solar pavement with micro photovoltaic array. Sustain. Energy Technol. Assessments.

[37] (2017). Italian Government Criteri Ambientali Minimi per l’edilizia.

[38] Chen X., Yang F., Zhang S., Zakeri B., Chen X., Liu C., Hou F. (2021). Regional emission pathways, energy transition paths and cost analysis under various effort-sharing approaches for meeting Paris agreement goals. Energy.

[39] Moretti L., Cantisani G., Carpiceci M., D’andrea A., Del Serrone G., Di Mascio P., Loprencipe G. (2021). Effect of sampietrini pavers on urban heat Islands. Int. J. Environ. Res. Publ. Health.

[40] Moretti L., Cantisani G., Carpiceci M., D'Andrea A., Del Serrone G., Di Mascio P., Peluso P., Loprencipe G. (2022). Investigation of parking lot pavements to counteract urban heat Islands. Sustainability.

[41] Del Serrone G., Peluso P., Moretti L. (2022). Evaluation of microclimate benefits due to cool pavements and green infrastructures on urban heat Islands. Atmosphere.

[42] Alsaad H., Hartmann M., Hilbel R., Voelker C. (2022). ENVI-met validation data accompanied with simulation data of the impact of facade greening on the urban microclimate. Data Br.

[43] Acharya T., Riehl B., Fuchs A. (2021). Effects of albedo and thermal inertia on pavement surface temperatures with convective boundary conditions—a CFD study. Processes.

[44] de Morais M.V.B., Guerrero V.V.U., de Freitas E.D., Marciotto E.R., Valdés H., Correa C., Agredano R., Vera-Puerto I. (2019). Sensitivity of radiative and thermal properties of building material in the urban atmosphere. Sustain. Times.

[45] Qin, Y.; Zhang, · Xingyue; Tan, K.; Wang, J. A Review on the Influencing Factors of Pavement Surface Temperature 1, 3, doi:10.1007/s11356-022-22295-3.. 10.1007/s11356-022-22295-3 35931844

[46] Asdrubali F., D'Alessandro F., Baldinelli G., Bianchi F. (2014). Evaluating in situ thermal transmittance of green buildings masonries—a case study. Case Stud. Constr. Mater..

[47] Tseliou A., Koletsis I., Pantavou K., Thoma E., Lykoudis S., Tsiros I.X. (2022). Evaluating the effects of different mitigation strategies on the warm thermal environment of an urban square in athens, Greece. Urban Clim..

[48] Tsoka S., Tsikaloudaki A., Theodosiou T. (2018). Analyzing the ENVI-met microclimate model's performance and assessing cool materials and urban vegetation applications–A review. Sustain. Cities Soc..

[49] Fabbri K., Di Nunzio A., Gaspari J., Antonini E., Boeri A. (2017). Outdoor comfort: the ENVI-BUG tool to evaluate PMV values output comfort point by point. Energy Proc..

[50] https://www.ilmeteo.net/meteo_Roma-Europa-Italia-Roma--sactual-31010.html.

[51] Bruse M., software H.F.-E., modelling (1998).

[52] Roth M., Environment V.L.-B. (2017).

[53] Romana D’ F., Alfano A., Ficco G., Frattolillo A., Palella B.I., Riccio G. (2021). Mean radiant temperature measurements through small black globes under forced convection conditions. mdpi.com.

[54] Luki M., Filipovi D., Pecelj M., Crnogorac L., Luki B., Divjak L., Luki A., Vuči A., Jänicke B., Dzyuban Y. (2021). Assessment of outdoor thermal comfort in Serbia's urban environments during different seasons. mdpi.com.

[55] UNI (Ente Italiano di Normazione) (2006).

[56] in, P.F.-T. comfort. A. and applications (1970). Undefined Thermal Comfort. Analysis and Applications in Environmental Engineering.

[57] (2017). ASHRAE Standard 55-2017; Thermal Environmental Conditions for Human Occupancy.

[58] Ma T., Li S., Gu W., Weng S., Peng J., Xiao G. (2022). Solar energy harvesting pavements on the road: comparative study and performance assessment. Sustain. Cities Soc..

[59] Zhou B., Pei J., Nasir D.M., Zhang J. (2021). A review on solar pavement and photovoltaic/thermal (PV/T) system. Transp. Res. Part D Transp. Environ..

[60] JRC Photovoltaic Geographical Information System (PVGIS) - European Commission Available online: https://re.jrc.ec.europa.eu/pvg_tools/en/(accessed on 15 February 2023).

[61] http://Www.Dial.

[62] Fotios S., Boyce P., Ellis C. (2005).

[63] (2014). European Standard EN 12464–2:2014 Light and Lighting—Lighting of Work Places—Part 2: Outdoor Work Places.

[64] ARERA - Modalità per l’attribuzione Su Base Oraria Dell’energia Elettrica Prelevata Dagli Impianti Di Illuminazione Pubblica Available online: https://www.arera.it/it/docs/04/052-04.htm (accessed on 15 February 2023).

[65] Dezfooli A.S., Nejad F.M., Zakeri H., Kazemifard S. (2017). Solar pavement: a new emerging technology. Sol. Energy.

[66] Ahmad A.F., Razali A.R., Romlay F.R.M., Razelan I.S.M. (2021). Energy harvesting on pavement A review. International Journal of Renewable Energy Research (IJRER).

[67] Liu Z., Yang A., Gao M., Jiang H., Kang Y., Zhang F., Fei T. (2019). Towards feasibility of photovoltaic road for urban traffic-solar energy estimation using street view image. J. Clean. Prod..

[68] Fakour H., Imani M., Lo S.L., Yuan M.H., Chen C.K., Mobasser S., Muangthai I. (2023). Evaluation of solar photovoltaic carport canopy with electric vehicle charging potential. Sci. Rep..

[69] Deshmukh S.S., Pearce J.M. (2021). Electric vehicle charging potential from retail parking lot solar photovoltaic awnings. Renew. Energy.

[70] He H., Li S., Wang S., Li B., Zhao J., Ma F. (2022). Investment strategies under stochastic electricity prices and implications for charging infrastructure network coverage: a case of photovoltaic pavements. Comput. Ind. Eng..

